# Future Perspectives on Targeting the Activated TLR4/NFκB Pathway in Cystic Fibrosis: A Possible Interplay Between Ethnopharmacology and microRNA Therapeutics

**DOI:** 10.3390/molecules30214155

**Published:** 2025-10-22

**Authors:** Roberto Gambari, Alessia Finotti

**Affiliations:** Department of Life Sciences and Biotechnology, Ferrara University, I-44121 Ferrara, Italy

**Keywords:** toll-like receptor 4, nuclear Factor-κB, natural products, garlic, micro RNAs

## Abstract

Cystic fibrosis (CF) is an inherited genetic disease caused by dysregulation of the cystic fibrosis transmembrane regulator (CFTR) gene, a chronic hyperinflammatory state and frequently occurring severe bacterial infections of the lungs. Novel protocols for treating CF inflammation are highly needed. Among the most interesting fields of pre-clinical investigation, the use of natural products, including those used in ethnopharmacology, appears to be promising. Examples of natural ethnopharmacological products that should be further investigated as potential anti-inflammatory agents for CF include inhibitors of the Toll-like receptor 4 (TLR4)/Nuclear Factor κB (TLR4/NFκB) pathway, such as parthenolide, curcumin and garlic-related constituents. In addition, “miRNA therapeutics” protocols have been reported as able to dampen the expression of pro-inflammatory genes. These two fields of investigation deserve, in the near future, further experimental efforts. Notably, these two approaches can be combined in order to develop novel strategies to improve the inhibitory activity on the expression of key pro-inflammatory genes activated in cystic fibrosis, including those induced by *Pseudomonas aeruginosa* infection.

## 1. The Toll-like Receptor 4 (TLR4)/NFκB Signaling Pathway in Cystic Fibrosis

**Figure 1:**
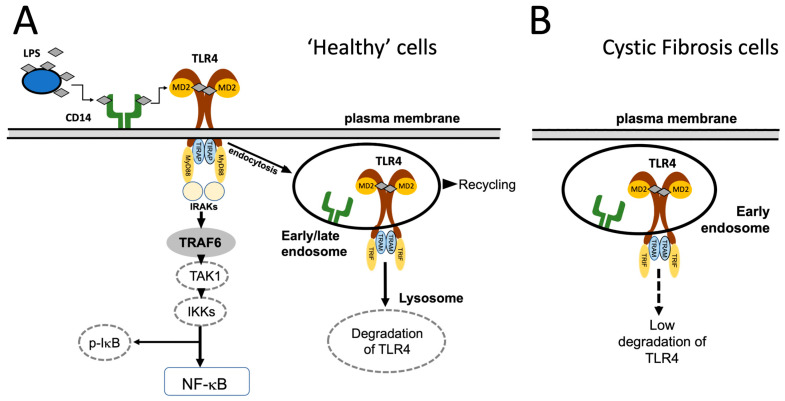
(**A**) The LPS-dependent activation of the TLR4/NFκB pathway and the TLR4 endocytosis and lysosomal degradation in “healthy” cells. (**B**) In cystic fibrosis cells, TLR4 is not efficiently targeted to the lysosomal compartment, leading to low degradation. This pictorial representation of the TLR4/NFκB pathway is inspired by the studies published by Bruscia et al. [10], Ciesielska et al. [12], Halaas et al. [13] and Kelly et al. [14]. Modified from Ciesielska et al. [12] with permission (copyright can be found at: https://link.springer.com/article/10.1007/s00018-020-03656-y#rightslink; accessed on 26 September 2025).

Cystic fibrosis (CF) is an inherited genetic disease caused by the following clinical/biochemical features: (a) dysregulation of the cystic fibrosis transmembrane regulator (CFTR) gene, (b) a chronic hyperinflammatory state and (c) frequent and severe bacterial infections of the lungs [1,2,3,4]. This important genetic disease has been the object of a relevant number of investigations, supporting the general concept that, as expected, understanding the molecular basis of the disease is a key step to develop tailored therapies, as reviewed by Rey et al. [5]. In this respect, the Toll-like Receptor 4 (TLR4)/NFκB pathway is involved in the pathogenesis of CF and is a potential target of therapeutic interventions [6,7,8]. In *Pseudomonas aeruginosa* (*P. aeruginosa*) infected cells, TLR4 recognizes *P. aeruginosa* components, such as lipopolysaccharide (LPS), activating the downstream NFκB signaling pathways. NFκB activation sustains the NFκB-dependent excessive expression of pro-inflammatory genes that is a recognized characteristic of CF [9]. Notably, and of great relevance for CF, Bruscia et al. reported an abnormal trafficking and degradation of TLR4 in cystic fibrosis [10]. When “healthy” cells are compared with cystic fibrosis cells, relevant differences have been reported with respect to the TLR4 pathway, as shown in Figure 1. In “healthy” cells, the stimulation by bacterial LPS triggers TLR4, activating the MyD88-dependent signaling. TLR4 is then internalized, processed and undergoes degradation in the lysosomal compartment (Figure 1A). These processes are finely tuned and are the basis for a regulated and self-limiting inflammatory response [11]. In cystic fibrosis cells, the loss of functional CFTR impairs the normal endocytic and lysosomal trafficking of TLR4, which remains in early endosomes, failing to be degraded (Figure 1B) [7,12,13,14]. In this respect, Kelly et al. have reported a study demonstrating that TLR4 is not targeted to the lysosomal compartment in cystic fibrosis airway epithelial cells [14]. This abnormal TLR4 trafficking causes a further increased activation of the NFκB pathway in CF [15]. These concepts have been extensively discussed elsewhere [10,12,13,14] and are summarized in Figure 1.

## 2. Therapeutic Relevance of Inhibitors of the TLR4/NFκB Pathway in Cystic Fibrosis

Targeting the TLR4/NFκB pathway with inhibitors could be a promising approach to reduce inflammation [16,17]. In CF, this effect might be associated with improvement of lung function and mitigation of other clinical complications in CF patients. Review articles reporting the development and validation of low-molecular weight inhibitors of the TLR4/NFκB pathway are available [18,19,20,21]. In this section, a few examples of such inhibitors are reported, briefly discussing their employment in ethnopharmacology and possible application in pre-clinical research on cystic fibrosis. Notably, as discussed by Patwardhan and Aswar, the modern impact of ethnopharmacology is high and concerns overcoming key challenges in drug discovery (such as long drug development times, very high costs, high risk of failure and possible adverse drug reactions) [22]. A further consideration is that products used in ethnopharmacology could be combined with innovative technologies (e.g., those based on nucleic acids) to drive cutting-edge therapeutic developments [22].

### 2.1. TAK-242

TAK-242 (resatorvid) is a cyclohexane selected for inhibition of TLR4 [23]. There is general agreement on the fact that, among 10 different human TLRs, TAK-242 selectively binds to TLR4. TAK-242 binds to the cysteine residue 747, preventing TLR4 binding with the toll-interleukin-1 receptor (TIR) domain-containing adaptor protein (TIRAP) [23,24] and downstream signal transduction. This mechanism of action makes TAK-242 a therapeutic candidate for conditions driven by excessive TLR4 signaling, such as sepsis and rheumatoid arthritis [25,26]. In this respect, TAK-242 has been used for mitigating experimental fibrosis and inflammation [23,26], suggesting potential applications in conditions such as cystic fibrosis. However, studies on the possible use of TAK-242 for the treatment of CF are lacking, and research efforts on this issue are highly warranted and needed.

### 2.2. Parthenolide

Parthenolide (PTL) is a sesquiterpene lactone naturally found in the feverfew plant (*Tanacetum parthenium*), which has been used for centuries as an herbal remedy in folk medicine for its anti-inflammatory properties [27]. PTL can be therefore considered as a “natural product of ethnopharmacological origin” [28,29]. Parthenolide is a strong inhibitor of the TLR4/NFκB pathway, as suggested by Park et al., who reported that PTL inhibits the TRIF-dependent signaling pathway of Toll-like receptors, including TLR4 [30]. Biochemical evidence indicates that PTL blocks both the MyD88 and TRIF branches of the TLR4 signaling pathway [30,31]. Accordingly, Li et al. found that parthenolide inhibited LPS-induced inflammatory cytokines in THP-1 cells [31]. With respect to possible treatment of the hyperinflammatory conditions in cystic fibrosis, “in vitro” experiments using CF cell lines and “in vivo” studies on CFTR-knockout mice demonstrated that PTL reduces inflammation by inhibiting NFκB activation, thereby decreasing the production of pro-inflammatory mediators, such as IL-8, the most relevant chemokine in CF [32]. Although current research on parthenolide for cystic fibrosis is primarily preclinical, its ability to potentially combat the chronic inflammation characteristic of CF-associated lung disease makes it a promising candidate for future anti-inflammatory treatments.

### 2.3. Sulforaphane

Sulforaphane (SFN) is a major component of *Brassica Oleracea* [33], retaining several biological activities, including activation of Nfr2 (a key regulator of anti-oxidant defense), reduction of oxidative stress, anti-fibrotic activity, and reduction of inflammation [34,35]. Regarding the SFN mechanism of action, Koo et al. have reported that this bioactive compound inhibits LPS engagement with the TLR4/MD2 complex by preferential binding to Cys133 in the hydrophobic pocket of MD2 [36]. Accordingly, Wang et al. have published a study showing that SFN inhibits LPS-induced changes in treated macrophages by altering the TLR4/NFκB pathway, suggesting SFN treatment as a possible therapeutic strategy for acute lung injury (ALI) [37]. Taken together, these findings support the concept that SFN might hold strong potential in inhibiting CF hyperinflammatory conditions through downregulation of the TLR4/NFκB signaling pathway.

### 2.4. Curcumin

Curcumin is extracted from *Curcuma longa* and is a constituent (up to ∼5%) of the traditional medicine known as turmeric [38,39]. The ethnomedicinal uses, phytochemistry, pharmacological activities and toxicity profiles of curcumin are well known and recognized, as reported in several review papers [40,41,42]. Interest in the therapeutic use of turmeric and the relatively easy isolation of curcuminoids has led to their extensive investigation [39]. Notably, curcumin exerts its anti-inflammatory activity through inhibition of the TLR4/NFκB signaling pathway [43,44,45]. Research articles reported that curcumin inhibited inflammation in human bronchial epithelial cells [46] and reduced inflammation-associated tissue damage in a preclinical model of CF [47].

### 2.5. Trimethylangelicin

The psoralen-related compound 4,6,4′-trimethylangelicin (TMA) potentiates the activation of wild-type CFTR [48] and rescues F508del-CFTR-dependent chloride secretion in both primary and secondary airway cells homozygous for the F508del CF mutation [48]. It was recently demonstrated that TMA, like lumacaftor (VX-809), stabilizes the first membrane-spanning domain (MSD1) and enhances the interface between NBD1 and ICL4 (MSD2) [49]. TMA also demonstrated anti-inflammatory properties, via reduction of IL-8 expression, thus making TMA (and TMA analogues exhibiting low side effects) promising agents for treatment of cystic fibrosis [49]. Notably, a docking site of TMA into NFκB was reported based on in silico analysis, strongly suggesting NFκB as a potential molecular target of TMA [50]. To further understand whether the NFκB pathway should be considered a target of TMA, chromatin immunoprecipitation (ChIP) was performed, observing that TMA preincubated in whole living cells reduced the interaction of NFκB with the promoter of the IL-8 gene; these results suggest that TMA could inhibit IL-8 gene transcription by intervening in the recruitment of NFκB on the IL-8 gene promoter [48]. The interest in TMA and TMA analogues is further demonstrated by the orphan designation, which was granted by the European Commission to Rare Partners srl Impresa Sociale (Italy), for the treatment of cystic fibrosis with 4,6,4′-trimethylangelicin (EU/3/13/1137). Furthermore, the TMA analogue GY971 was recently designated as an anti-inflammatory orphan drug by the European Medicines Agency (EMA) on 10 November 2024 for the treatment of cystic fibrosis (https://www.ema.europa.eu/en/medicines/human/orphan-designations/eu-3-24-2976; accessed on 5 September 2025).

### 2.6. JSH-23

4-Methyl-N1-(3-phenyl-propyl)-benzene-1,2-diamine (JSH-23) is a novel chemically synthetic compound. This compound exhibited inhibitory effect with an IC_50_ value of 7.1 μM on NFκB transcriptional activity in LPS-stimulated RAW 264.7 macrophages, interfering with LPS-induced nuclear translocation of NFκB without affecting IκB degradation [51]. Furthermore, the compound inhibited not only LPS-induced expression of TNF-α, IL-1β, IL-6, inducible nitric oxide synthase and cyclooxygenase-2, but also LPS-induced apoptosis of the RAW 264.7 cells. In this respect, Kelly et al. studied the effects of JSH-23 on LPS-stimulated CF cells. After LPS stimulation, they found a decreased expression of A20, a cytoplasmic zinc finger protein inhibiting Toll-like receptor-activated NFκB signaling by deubiquitinating TRAF-6. Consequently, NFκB was activated, and the release of IL-8 strongly induced. Treatment with JSH-23 fully suppressed NFκB activation and IL-8 release [52]. Compounds showing inhibitory activities against the NFκB pathways, such as JSH-23, might be considered promising pre-clinical agents to develop clinically oriented protocols to control the hyperinflammatory state of cystic fibrosis.

### 2.7. Aged Garlic Extract and Its Constituents S-Allyl-Cysteine (SAC) and S1-Propenyl-Cysteine (S1PC)

**Figure 2:**
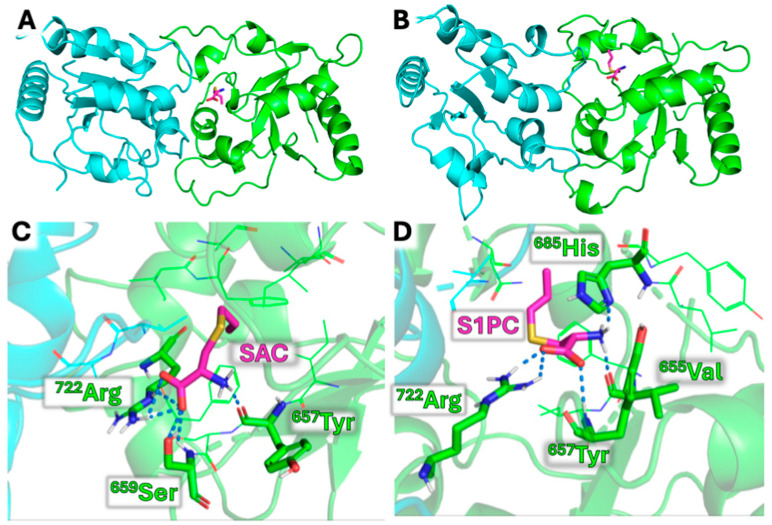
Results of docking simulations. (**A**,**B**). Binding mode predicted for SAC (**A**) and S1PC (**B**) with the TLR4-TIR dimer. (**C**,**D**). Details of predicted interactions of SAC (**C**) and S1PC (**D**). Hydrogen bonds are depicted as dashed blue lines. This pictorial representation is based on the results reported by Gasparello et al. [59] and Papi et al. [60]. Copyrights related to these two articles can be found at https://www.mdpi.com/1420-3049/29/24/5938 and https://pmc.ncbi.nlm.nih.gov/articles/PMC12171798/ (accessed on 26 September 2025).

Among a large variety of natural products of biomedical relevance interfering with the TLR4/NFκB pathway, garlic (*Allium sativum*)-based products have recently gained great attention [53,54]. The impact of garlic in ethnopharmacology has been recently discussed by Yaniv Bachrach [55] and by Tudu et al. [56]. According to the study published by Yaniv Bachrach, garlic has been an integral part of human culture and medicine for over 5000 years, serving as both a food supplement and a therapeutic agent. The ethnobotanical and medicinal significance of garlic across civilizations, from its origins in Central Asia to its worldwide dissemination, is demonstrated by the fact that ancient cultures, including those of Egypt, Greece, China, Persia, Sumer and India, integrated garlic into diets, rituals and medicinal practices, recognizing its biological properties and health benefits [55]. Among garlic-related products, aged garlic extract (AGE) is well known and has been studied in detail [57]. The beneficial effects of AGE have been attributed to several bioactive compounds, including lipid-soluble allyl sulfur compounds (e.g., diallyl sulfide, diallyl disulfide and diallyl trisulfide) and water-soluble compounds, such as S-allyl-cysteine (SAC), S-allylmercaptocysteine (SAMC) and S1-propenyl-cysteine (S1PC) [58]. Interestingly, the AGE components SAC and S1PC bind TLR4, as depicted in Figure 2 [59,60,61], and inhibit NFκB, as found in several studies performed using different methodological approaches and different cellular model systems, such as bronchial epithelial cells [59,60], chondrocytes [61] and T lymphoid cells [62]. This biomolecular mechanism of action of SAC and S1PC might explain the diverse biological effects of these compounds, including the anti-inflammatory activity. Interestingly, the pharmacokinetic profile (absorption, distribution, metabolism and excretion) of SAC and S1PC is known and characterized by rapid absorption after oral administration and high bioavailability (88.0–95.8% in rats, mice and dogs) [63,64]. SAC and S1PC exhibit high and relatively long-lasting plasma concentrations and are excreted in urine in their N-acetylated forms [65]. This information might contribute to pre-clinical studies focusing on biomedical applications of these compounds.

The information on the effects of AGE, SAC and S1PC on cystic fibrosis is still lacking, although inhibition of TLR4/NFκB-dependent expression of pro-inflammatory genes might occur and should be considered [59,60]. Figure 2 shows a molecular docking study indicating that SAC and S1PC are able to interact with TLR4. When combined with molecular dynamics (MD) studies, the data obtained sustain the hypothesis that the binding of SAC and S1PC might lead to a destabilization of the TIR-TLR4 dimer, and that this effect can be the leading cause of their anti-inflammatory effect [59,60].

### 2.8. Other Examples of Natural Products Affecting the TLR4/NFκB Pathway

Several natural products isolated from medicinal plants deserve attention and pre-clinical studies in the field of therapeutic intervention for cystic fibrosis using inhibitors of the TLR4/NFκB pathway. A few examples are isoliquiritigenin isolated from the roots of *Glycyrrhiza uralensis* [66], eugenolol isolated from *Syzygium aromaticum*, *Cinnamomum verum*, and *Laurus nobilis* [67], euscaphic acid isolated from the roots of *Rosa rugosa* [68], and cnidilide, isolated from the roots of *Cnidium officinalis* [69]. Detailed information on natural products interfering with the TLR4/NFκB pathway has been published by Li et al. [70] and Zhao et al. [71], among others.

## 3. MicroRNA Therapeutics and Inhibitory Effects on the TLR4/NFκB Pathway

Micro RNAs are a class of small, single-stranded, non-coding RNAs that function as a guide molecule in RNA silencing and hence modulate gene expression [72,73,74,75,76,77]. The discovery of the deep involvement of miRNAs in several human pathologies has generated interest in pre-clinical studies, demonstrating the possible application of the so-called “MicroRNA Therapeutics” for the development of clinical protocols [78,79,80]. Several review articles are available describing the biogenesis of miRNAs, their processing, and the mechanisms of translational suppression or degradation of target mRNAs [74,75,76,77].

### 3.1. MicroRNAs Targeting the TLR4/NFκB Pathway

A microRNA regulatory network has been reported to regulate the NFκB signaling pathway [81]. In this study, miR-146a-mediated repression of NFκB activation was reported in mouse macrophages. Usually, microRNAs regulating the TLR4/NFκB pathway bind to mRNAs coding downstream signaling molecules, causing promotion or inhibition of the inflammatory process [82,83]. Examples of microRNAs affecting the TLR4/NFκB pathway are miR-16 [84], miR-93 [85], the already cited miR-146a [81,86], miR-329 [87], miR-489 [88] and Let-7b [89]. Concerning therapeutic protocols, miRNAs regulating the TLR4/NFκB pathway might be considered as potential therapeutic agents for diseases caused by chronic inflammation.

In this respect, the feasibility of the “miRNA-mimicking” approach is strongly supported by the fact that clinical trials sponsored by pharmaceutical companies, such as MiRagen Therapeutics and EnGene IC, have been designed. Examples of the trials were those based on mimicking the activity of miR-29 (NCT02603224) [90], miR-34a (NCT01829971) [91] and miR-16 (NCT02369198) [92]. Notably, miR-16 has been shown to affect the TLR4/NFκB pathway [84].

### 3.2. MicroRNA Therapeutics Based on Transfection with Pre-miR93-5p: Potential Effects on the TLR4/NFκB Pathway

**Figure 3:**
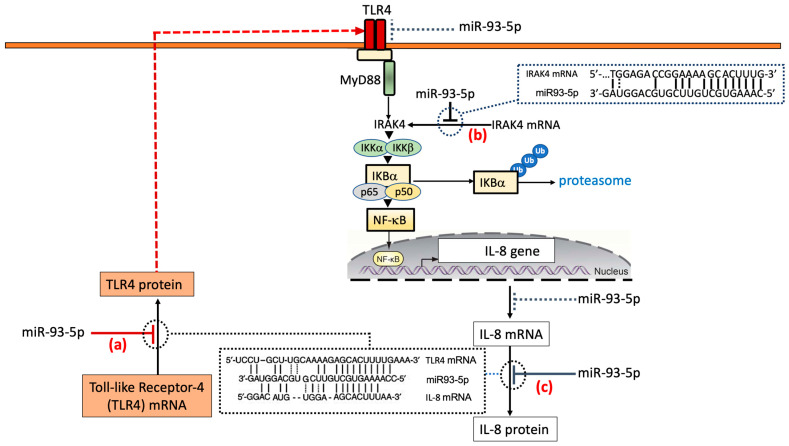
Binding of miR-93-5p to TLR4 (**a**), IRAK4 (**b**) and IL-8 (**c**) mRNAs: effects on the TLR4/NFκB pathway and IL-8 production. This proposed mechanism of action is based on the studies published by Fabbri et al. [85], Gao et al. [93], Xu et al. [94], and Tian et al. [95].

Figure 3 shows the extent of complementarity between miR-93-5p and the 3′-UTRs of TLR4 (Figure 3a), IRAK4 (Figure 3b) and IL-8 (Figure 3c) mRNAs. Considering TLR4 and IL-8 mRNAs, it should be noted that the number of hydrogen bonds generated between miR-93-5p and the 3′-UTRs of TLR4 and IL-8 mRNAs are very similar, suggesting that miR-93-5p directly targets both TLR4 and IL-8 mRNAs. In this respect, the study published by Gao et al. [93], employed a suitably delivered luciferase construct, composed of a partial sequence of the TLR4 3′-UTR (encompassing the binding sites of miR-93) sub-cloned into a dual-luciferase reporter vector [93]. The results obtained demonstrated that transfection of RAW264.7 cells with miR-93 mimics inhibited the luciferase activity directed by the TLR4-3′UTR reporter plasmid. On the contrary, co-transfection of the cells with an miR-93 inhibitor enhanced luciferase activity. Similar results were obtained by Fabbri et al. [85] using a vector carrying the 3′-UTR of human IL-8 mRNA. The studies by Gao et al. [93] and Fabbri et al. [85] indicated a direct interaction of miR-93-5p with IL-8 and TLR4 mRNAs, in accordance with the model depicted in Figure 3.

Notably, miR-93-5p could significantly affect NFκB signaling by a direct interaction with IL-1 receptor-associated kinase 4 (IRAK4) mRNA, as depicted in Figure 3b. Interestingly, IRAK4 activates NFκB [94,95]. Summarizing, in the theoretical model depicted in Figure 3, the IL-8 expression level is directly inhibited by miR-93-5p (Figure 3c), as suggested by Fabbri et al. [85]. Additionally, IL-8 expression could be indirectly suppressed through the inhibition of the TLR4/NFκB pathway. This is caused by the direct miR-93-5p-mediated TLR4 downregulation [93] and the direct interaction (and consequent inhibition) with IRAK4 mRNA [94,95].

## 4. MicroRNA Therapeutics in Combination with Garlic-Based Ethnopharmacology: Future Perspectives in the Development of Pre-Clinical Protocols for Cystic Fibrosis

Considering the TLR4/NFκB signaling pathway as a potential target of anti-inflammatory protocols for cystic fibrosis, future perspectives should include pre-clinical studies based on natural products (as described in Section 2) and microRNA therapeutics (as described in Section 3 and extensively discussed by Finotti and Gambari) [96]. Among the several natural products, those based on garlic should be considered, in our opinion, with great interest, considering the increasing number of publications available on their anti-inflammatory and anti-oxidant potential, the increasing number of patents and patent applications, and the ongoing clinical trials [97]. In addition to protocols based on natural products, future perspectives on therapeutic interventions for cystic fibrosis should include protocols based on microRNAs interfering with the TLR4/NFκB signaling pathway, such as miR-93-5p. Transfection of cystic fibrosis cells with pre-miR-93-5p might inhibit the expression of pro-inflammatory genes regulated through the TLR4/NFκB pathway.

**Figure 4:**
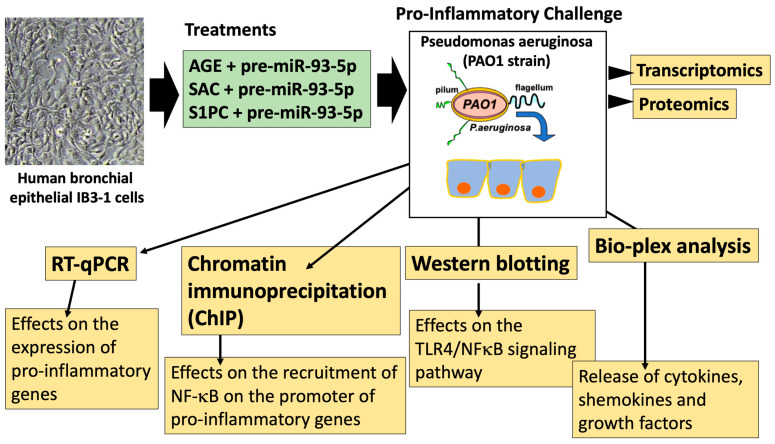
A proposed experimental strategy to verify the potential of combined treatments of PAO1-infected cystic fibrosis IB3-1 cells with natural products (AGE, SAC and S1PC) and pre-miR-93-5p (treatments are indicated in the green box). Proposed endpoints are indicated in the yellow boxes.

Notably, the two approaches (one based on natural products, the other based on pre-miRNAs) can be combined in order to develop novel strategies to improve the interference with the TLR4/NFκB pathway and consequent inhibition of inflammation based on a transcriptional decrease in the expression of pro-inflammatory genes. This experimental strategy has been previously discussed [98] and is depicted in Figure 4.

In this example, cystic fibrosis bronchial epithelial IB3-1 cells are employed and treated with natural products (AGE, SAC or S1PC) administered singularly or in combination with pre-miR-93-5p. This pre-clinical model might be considered, in our opinion, a priority to guide future research and to reach a proof-of-concept to be confirmed in other, similar experimental CF model systems, including in vivo models. Regarding the proposed endpoints, gene expression might be evaluated by RT-qPCR and Western blotting in order to examine the effects of the treatments on the TLRNFκB pathway and on the expression of pro-inflammatory genes (such as IL-1β, IL-6, IL-8 and other NFκB-regulated genes). In addition, other highly warranted endpoints are based on transcriptomic and proteomic studies. The effects of the treatments on the recruitment of NFκB to the promoters of NFκB-regulated genes could be studied by chromatic immunoprecipitation (ChIP), as described by Tamanini et al. [48] using monoclonal antibodies against NFκB.

Several considerations should be made on the experimental approach described in Figure 4. First of all, considering that reliance on single studies reduces critical depth, it is required to confirm the results obtained using the IB3-1 cells (Figure 4) with results obtained with other “in vitro” and “ex vivo” experimental model systems. Examples of available CF cellular model systems are the CFBE41o- cells and CFTE29o- cells, which are human bronchial and tracheal epithelial cells with CFTR mutations [99,100]. Concerning primary cells, although primary human bronchial epithelial (HBE) cells are the “gold-standard” for drug discovery in cystic fibrosis research, a number of other cell models with different CF genotypes have been developed, including CRISPR/Cas9 gene-edited HBE. Non-human cell lines are also available, such as Fischer Rat Thyroid Cells (FRT) [99]. Confirmation of the results using different cellular model systems should be considered a strength of the proposed research activity.

Second, the fact that only garlic constituents and only the pre-miR-93-5p sequence are considered in the experimental plan shown in Figure 4 is certainly an important limit. Therefore, in order to overcome this limit, additional natural products (some examples are described in Section 2) and pre-miRNAs (see Section 3) might be considered. This, on one hand, could facilitate comparison of the efficacy of the treatments, and on the other hand, will allow finding the most active anti-inflammatory combinations when combined treatments are performed. As described in Section 2, while several natural products (such as TAK-242, curcumin, parthenolide, JAK-23 and sulforaphane) are potent inhibitors of the TLR4/NFκB pathway, their activity on pro-inflammatory genes expressed in cystic fibrosis experimental model systems has not been studied so far in detail. In our opinion, in addition to garlic-related products, priority should be given to natural products already used in ethnopharmacology, such as curcumin and parthenolide (for which low costs, high efficacy and low side effects are expected) [22]. As far as micro-RNA therapeutics, in addition to pre-miR-93, the effects of other pre-miRNAs targeting the TLR4/NFκB signaling pathway (see Section 3) should be studied on cystic fibrosis experimental model systems, also combined with anti-inflammatory natural products.

In this respect, the novel hypothesis emerging from the studies discussed here is that molecules developed within the “microRNA Therapeutic approach” and natural products used in ethnopharmacology can be combined for the treatment of cystic fibrosis. To the best of our knowledge, this interesting and original combined strategy has not been analyzed in depth. Despite the fact that this approach is highly speculative, a first preliminary proof-of-concept experiment has been recently reported, demonstrating that the combined treatment of IB3-1 cells with AGE + pre-miR-93-5p is associated with the highest level of inhibition of induced IL-8 gene expression, compared with singularly added AGE or pre-miR-93-5p [98].

To fully verify the translational potential of the combined approach, several challenges need to be considered, such as delivery of natural products and pre-microRNAs, tissue distribution in the context of such a complex disease as cystic fibrosis. Regarding these specific points, it should be considered that the delivery systems for efficient targeting of the lung epithelium need to overcome the mucus barriers and the microbial biofilms, both present in CF patients and possibly hindering the delivery and efficacy of nucleic acid-based therapies. In addition, we should consider that natural products and nucleic acids belong to very different categories of biomedical agents, with different delivery requirements and pharmacokinetic properties. The issue of delivery of nucleic acid-based biomolecules has been considered in a recent review paper, which reported the use of lipid nanoparticles (LNPs) for efficient delivery to the lung [101,102]. A comparative description of advanced formulations for pulmonary drug delivery in the treatment of cystic fibrosis is available [103].

Regarding feasibility, we would like to emphasize that (a) several pharmaceutical companies have been engaged in the “miRNA-mimicking” approach, as reviewed by Finotti and Gambari [96], and (b) clinical trials have been designed (for example, NCT02603224, NCT01829971 and NCT02369198) [90,91,92]. In those clinical trials, the issues of potential side effects and delivery strategies have been addressed. Concerning the feasibility and therapeutic potential of natural products already used in ethnopharmacology, they are expected to exhibit low costs, high efficacy and low levels of side effects [22]. Based on these considerations, the combined approach proposed here appears to be promising for the development of novel therapeutic protocols for cystic fibrosis.

## 5. Conclusions

Future perspectives in the development of new anti-inflammatory protocols for CF include pre-clinical studies based on agents able to interfere with the TLR4/NFκB signaling pathway, including promising natural products (such as aged garlic extracts and their constituents S-allyl-Cysteine and S1-propenyl- cysteine) and miRNA-based therapeutic biomolecules targeting the TLR4/NFκB pathway. In this respect, pre-miR-93-5p transfected to the cells is able to inhibit the TLR4/NFκB pathway by binding TLR4, IRAK4 and IL-8 mRNAs. Future perspectives in this challenging field of investigation should include studies based on the combination of natural products and miRNA-based molecules, to determine the impact of possible interplay between ethnopharmacology and microRNA therapeutics in the field of pre-clinical development of novel anti-inflammatory strategies for cystic fibrosis.

## Data Availability

No new data were created or analyzed in this study; additional information will be shared upon request to the corresponding authors.

## Abbreviations

The following abbreviations are used in this manuscript:

CF	Cystic Fibrosis
ALI	Acute Lung Injury
CFTR	Cystic fibrosis transmembrane regulator
TLR4	Toll-like Receptor-4
TIR	Toll-Interleukin-1 Receptor
TIRAP	TIR domain-containing adaptor protein
MyD88	Myeloid differentiation primary response 88
MD2	Myeloid differentiation factor 2
MSD1	First membrane-spanning domain
NBD1	Nucleotide-binding domain-1
ICL4	Four intracellular loops
NFκB	Nuclear Factor-kappa-B
Nfr2	Nuclear factor erythroid 2-related factor 2
TAK1	Transforming Growth Factor-β-activated kinase 1
IKK	IκB kinases
IKB	Nuclear factor of kappa light polypeptide gene enhancer in B-cells inhibitor
TRAM	Mal-related adaptor protein
TRIF	TIR domain-containing adaptor protein inducing interferon beta
TRAF6	TNF receptor associated factor 6
IRAK4	IL-1 receptor-associated kinase 4
IL	Interleukin
AGE	Aged Garlic Extract
SAC	S-allyl-l-cysteine
S1PC	S1-propenyl-l-cysteine
SAMC	S-allylmercaptocysteine
TMA	Trimethylangelicin
SFN	Sulforaphane
PTL	Parthenolide
JSH-23	4-Methyl-N1-(3-phenyl-propyl)-benzene-1,2-diamine
LPS	Lipopolysaccharide
PAO1	Pseudomonas aeruginosa O1 strain
miRNA	Micro RNA
UTR	Untranslated region
HBE	Human Bronchial Epithelial cells
FRT	Fischer Rat Thyroid cells
MD	Molecular Dynamics
ChIP	Chromatin Immunoprecipitation
RT-qPCR	Reverse Transcriptase-quantitative-PCR
EMA	European Medicines Agency
